# NLRP3 played a role in *Trichinella spiralis*-triggered Th2 and regulatory T cells response

**DOI:** 10.1186/s13567-020-00829-2

**Published:** 2020-08-27

**Authors:** Xuemin Jin, Xue Bai, Yong Yang, Jing Ding, Haining Shi, Baoquan Fu, Pascal Boireau, Mingyuan Liu, Xiaolei Liu

**Affiliations:** 1 Key Laboratory of Zoonosis Research, Ministry of Education, Institute of Zoonosis, College of Veterinary Medicine, Jilin University, Changchun, 130062 China; 2 Mucosal Immunology and Biology Research Center, Massachusetts General Hospital, Charlestown, MA USA; 3 Lanzhou Veterinary Research Institute, Chinese Academy of Agricultural Sciences, Lanzhou, China; 4 JRU BIPAR, ANSES, École Nationale Vétérinaire d’Alfort, INRA, Université Paris-Est, Animal Health Laboratory, Maisons-Alfort, France; 5 Jiangsu Co-innovation Center for Prevention and Control of Important Animal Infectious Diseases and Zoonoses, Yangzhou, Jiangsu People’s Republic of China

**Keywords:** *Trichinella spiralis*, excretory-secretory products, dendritic cell, NLRP3, Th2, Treg

## Abstract

*Trichinella spiralis* maintains chronic infections within its host. Muscle larvae excretory-secretory products (MLES) typically induce parasite-specific immune responses such as the Th2 response and regulatory T cells (Tregs) by modulating dendritic cell (DC) phenotype via the recognition of pattern recognition receptors (PRRs), such as Nod-like receptors (NLRs). We aimed to investigate the role of NLRP3 in *T. spiralis*-triggered immune response. We found that larvae burden was increased in NLRP3^−/−^ mice compared to wild type (WT) mice. Administration of MLES induced higher levels of IL-4, IL-10, TGF-β and population of Tregs in WT mice than in NLRP3^−/−^ mice. In vitro, we showed that increased expression of CD40 on the surface of MLES-treated DCs was inhibited after NLRP3 knockout. Increased production of IL-1β, IL-18, IL-10 and TGF-β, but not IL-12p70, was significantly diminished in the absence of NLRP3. Furthermore, our results demonstrated that MLES-treated DCs induced higher levels of IL-4, IL-10 and TGF-β and populations of Tregs in vitro. These inductions were abolished by NLRP3 deficiency in DCs, suggesting that NLRP3 in MLES-treated DCs plays a role in promoting the Th2 and Treg response. Taken together, we identified for the first time the involvement of NLRP3 in host defences against *T. spiralis*.

## Introduction

Parasitic diseases are a serious global health concern. Trichinellosis, caused by *Trichinella spiralis*, is one of the most prevalent neglected tropical diseases worldwide, and establishes chronic infection in a wide range of wild and domestic animals and human beings [1]. *T. spiralis* infection induces strong T helper 2 (Th2) immune response [2], which contributes equally to host defence against *T. spiralis* [3]. And Th2 cytokines production is regulated by regulatory T cells (Tregs) [4].

Parasite antigens have the potential to induce Th2 and Treg responses via dendritic cells (DCs). Indeed, DCs have numerous pattern recognition receptors (PRRs), such as Toll-like receptors (TLRs) or nucleotide-binding oligomerization domain (NOD)-like receptors (NLRs) [5]. *T. spiralis* muscle larvae excretory-secretory products (MLESs) enable the parasite to survive in the host by interacting with host immune cells and inducing an immune response [6]. *T. spiralis* MLESs direct the immunological balance away from Th1 cells towards Th2 cells and Tregs by modulating the DC phenotype via toll-like receptors (TLR) 2 and 4 [7]. NLRs signals are provided by TLR engagement that leads to the expression of inflammasome components [5]. TLR such as TLR4 accelerates the production of IL-1 family cytokines dependent on NLRs [8]. We therefore hypothesized that NLRs may play a role in modulating the function of *T. spiralis* MLESs-treated DCs.

The NLRP3 (NLR family, pyrin domain containing 3) inflammasome is composed of the protein NLRP3, the bipartite adaptor protein ASC (apoptosis-associated speck-like protein containing a CARD), and Caspase-1 and form an important arm of the innate immune system [9]. NLRP3 inflammasome complexes are the most intensively studied and are activated by a broad range of stimuli [10]. Interestingly, accumulating evidence indicates that NLRP3 activation is essential for the control of different parasitic infections. Activation of the NLRP3 inflammasome reduces *Toxoplasma gondii* infection load [11] and is critical for host resistance to diverse *Leishmania* species [12]. Inflammasomes are increasingly implicated in regulating immunity, but the relationship between their activation and the function of DCs remains largely unknown. It is still unclear whether NLRP3 in DCs is involved in the *T. spiralis*-induced immune response.

Here, we demonstrated that mice lacking NLRP3 but not ASC or Caspase-1/11 displayed significantly increased larval burdens. We therefore explored the role of NLRP3 in the immune response induced by *T. spiralis* infection. We first revealed that NLRP3 in DCs played a role in the induction of Th2 and Treg responses by MLES in vivo and in vitro via modulating DCs phenotype.

## Materials and methods

### Ethics statement

C57BL/6J wild-type (WT) mice (female, 4–6 weeks old) were purchased from the Norman Bethune University of Medical Science (NBUMS), China. Female Wistar rats were purchased from the Experimental Animal Centre of College of Basic Medical Sciences, Jilin University (Changchun, China). C57BL/6J NLRP3^−/−^, Caspase 1/11^−/−^, and ASC^−/−^ mice were kindly provided by Dr. Feng Shao. OT-II ovalbumin (OVA)-specific T-cell receptor (TCR) transgenic mice (on C57BL/6 background) were purchased from the Nanjing University Model Animal Research Centre (Nanjing, China). All animals were maintained on standard rodent chow with water supplied ad libitum under a 12 h/12 h light/dark cycle during the experimental period. All animal experiments followed the regulations on animal welfare and Public Health Service recommendations in China. All mice were handled strictly in accordance with the Animal Ethics Procedures and Guidelines of the People’s Republic of China. The protocol was approved by the Institutional Animal Care and Use Committee of Jilin University (protocol# 20170318).

### Parasites and preparation of MLES

The *T. spiralis* isolate (ISS534) was obtained from a naturally infected domestic pig in Henan Province in China. Briefly, Wistar rats were orally infected with 3000 infective larvae, and *T. spiralis* muscle larvae were recovered at 35 days post infection (dpi) via artificial digestion with pepsin-HCl (1% pepsin and 1% HCl at 37 °C for 2 h) [13]. All *T. spiralis* muscle larvae were washed three times in saline solution and incubated separately in prewarmed serum-free RPMI 1640 medium containing 2 mM l-glutamine, 100 U/mL penicillin, and 100 μg/mL streptomycin at 37 °C under 5% atmospheric CO_2_ for 24 h. After centrifugation, the supernatant containing ES products was dialyzed and concentrated [14]. According to the manufacturer’s instructions, the endotoxin was removed from the protein by using the ToxOut™ High Capacity Endotoxin Removal kit (Biovision, USA). There was about approximately 0.132 EU/mL residual endotoxin existing in MLES, approximately equivalent to 20 pg/mg endotoxin [15]. The protein concentration was determined by a Pierce bicinchoninic acid protein assay kit (Thermo Scientific, Rockford, IL). We made three different preparations of ES products from muscle larvae of *T. spiralis* to perform several independent experiments. One representative experiment is shown here.

### Larvae burden assessments

WT and NLRP3^−/−^ mice were infected with 300 muscle larvae (ML). MLs were recovered and counted at 35 dpi as previously described [16]. Briefly, the muscle tissues of infected mice were cut into pieces and digested by pepsin hydrochloric digestive fluid. The MLs were collected by washing three times in water with sedimentation and were counted with gelatine.

### Detection of MLES-induced immune response

WT and NLRP3^−/−^ mice were injected intraperitoneally (i.p.) with phosphate-buffered saline (PBS) or MLES (100 μg) twice at 7 days intervals. Spleen cells were isolated 7 days after second injection. Spleen cells were modulated to 1  ×  10^6^ cells/mL in complete RPMI-1640 with 10% fetal bovine serum (FBS), penicillin (100 U/mL) and streptomycin (100 μg/mL) and stimulated with MLES at a concentration of 50 μg/mL at 37 °C for 72 h in an incubator containing 5% CO_2_. The supernatants of spleen cells were harvested for detecting cytokines (IFN-γ, IL-4, IL-10 and TGF-β) production by ELISA (R&D Systems). The cells were resuspended in PBS for determining Tregs population by flow cytometry.

### Bone marrow-derived dendritic cell (BMDC) isolation, culture, and stimulation

Bone marrow-dendritic cells (BMDCs) were generated from mouse bone marrow cells as previously described [17]. Briefly, bone marrow cells were obtained from WT and NLRP3^−/−^ mice and cultured in RPMI 1640 medium containing 20 ng/mL recombinant GM-CSF, 20 ng/mL IL-4 (Sigma-Aldrich) and 10% FBS at 37 °C and 5% CO_2_. Immature DCs were enriched by positive selection with anti-CD11c magnetic beads (Miltenyi Biotec) according to the manufacturer’s instructions. DCs were harvested on day 7 for further experiments. To explore NLRP3 activation in MLES-treated DCs, the DCs were treated with MLES (50 μg/mL) in vitro for 24 h. Immature DCs were stimulated with sterile phosphate-buffered saline (PBS) as a control. Cells and cell culture supernatants were collected and stored at − 80 °C. Cytokines (IL-1β, IL-18, IL-12p70, IL-10 and TGF-β) levels in the supernatant were quantified by ELISA (R&D Systems). The stimulated DCs were stained with a PE-conjugated monoclonal antibody (mAb) to CD11c and APC-conjugated mAbs to CD40, CD80 or CD86 (Biolegend, USA). The cells were analysed by using a BD FACSCalibur Flow Cytometer and FlowJo software (Tree star Inc, Ashland, OR) [18].

### Co-culture of BMDCs with CD4^+^ T cells

CD4^+^ T cells from OT-II mice were purified from spleen cells by magnetic sorting using anti-CD4 magnetic beads (Miltenyi Biotec, Auburn, CA) as previously described [18]. The purified CD4^+^ T cells had > 90% purity. DCs (1 × 10^5^/well) were first treated with PBS or MLESs (50 μg/mL) for 24 h. DCs were washed three times with sterile PBS and resuspended in RPMI 1640 medium. DCs treated with PBS or MLESs and CD4^+^ T cells (1 × 10^6^/well) were cocultured with OVA (1 mg/mL) for 72 h. During the final 18 h, 2.5 μg/mL concanavalin-A (Con-A) (Sigma-Aldrich, St. Louis, MO, USA) was added. Samples were analysed using a BD FACSCalibur flow cytometer and FlowJo software (Tree star Inc, Ashland, OR). The supernatants of DC and CD4^+^ T cell cocultures were harvested for cytokine (IFN-γ, IL-4, IL-10 and TGF-β) analysis by ELISA kits (R&D Systems).

For T helper-related cytokines expression, after coculture of DCs (1 × 10^5^/well) and CD4^+^ T cells (1 × 10^6^/well) for 72 h with OVA (1 mg/mL), then cells were incubated with 10 mg/mL Brefeldin A (eBioscience, San Diego, CA, USA), 50 ng/mL phorbol 12-myristate 13-acetate (PMA) (eBioscience, San Diego, CA, USA) and 750 ng/mL Ionomycin (eBioscience, San Diego, CA, USA) for 6 h at 37 °C. Cells were stained for surface markers (FITC-anti-CD4 antibodies, BD Biosciences) for 35 min at 4 °C in the dark. These cells were fixed, permeabilized using a FIX/PERM set (Biolegend) and blocked in 5% rat serum for 10 min at room temperature prior to intracellular staining with APC-conjugated mAbs to IFN-γ, IL-4, IL-10 or TGF-β. In addition, the induction of Tregs by MLES-treated DCs was measured. CD4^+^ cells were first preincubated with Fc Blocker (anti-mouse CD16/CD32, BD Biosciences, USA) for 15 min to reduce nonspecific binding of labelled antibodies, then stained with APC-labelled anti-CD25, fixed and permeabilized using a FIX/PERM set (BD Biosciences, USA). The cells were then incubated in CD16/CD32 antibody prior to intracellular staining for Percp Cy5.5-labelled anti-Foxp3. For Foxp3 staining, the cells were fixed and permeabilized using the Foxp3 transcription factor staining buffer kit (eBioscience) according to the manufacturer’s instructions. Appropriately labelled isotype-matched antibodies were used as controls. Samples were analysed by using a BD FACSCalibur flow cytometer and FlowJo software (Tree star Inc, Ashland, OR).

### Statistical analysis

All results are expressed as the mean ± SD. Statistical analysis was performed using GraphPad Prism 5 for Windows. Two experimental groups were compared using Student’s t-test for nonparametric data. Three or more groups were compared through one-way analysis of variance (ANOVA) with Tukey’s multiple comparison test or Dunnett’s multiple comparison test as indicated. Two-way ANOVA with a Bonferroni test for multiple comparisons was used to compare the data. P values are expressed as *p < 0.05, **p < 0.01 and ***p < 0.001.

## Result

### *T. spiralis* larval burden increased in mice lacking NLRP3 but not Caspase 1/11 or ASC

**Figure 1:**
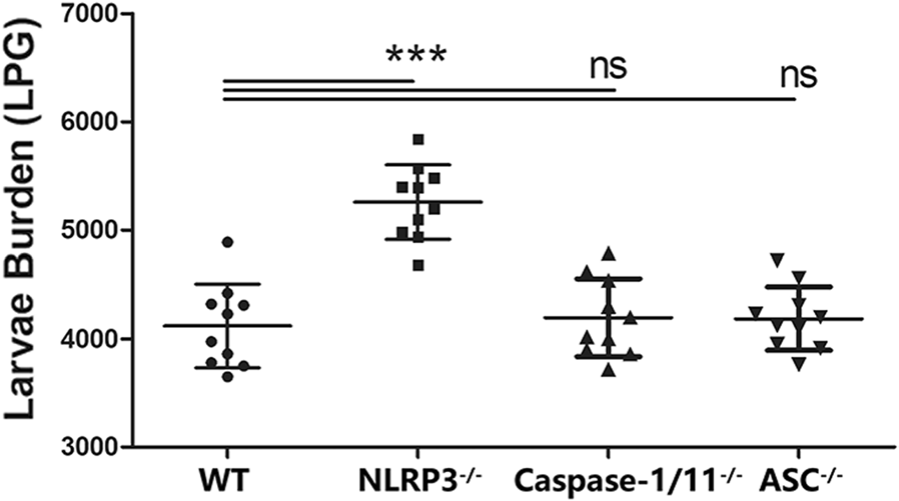
***T. spiralis*** **larval burden increased in mice lacking NLRP3 but not Caspase 1/11 or ASC**. WT and NLRP3^−/−^ mice were infected with 300 ML. Muscle larvae were recovered at 35 dpi from WT and NLRP3^−/−^ mice and calculated. The symbols represent individual animals, and horizontal lines indicate the medians. The data shown are representative of three independent experiments. *p < 0.05, **p < 0.01 and ***p < 0.001 vs WT mice (n = 10) as indicated by the line (Dunnett’s multiple comparison following ANOVA). No significance is marked as ns.

To determine whether NLRP3 inflammasome complexes, including NLRP3, Caspase 1 and ASC, played a role in the interaction between *T. spiralis* and the host, muscle larvae were recovered from WT, NLRP3^−/−^, Caspase 1/11^−/−^, and ASC^−/−^ mice infected with *T. spiralis* and quantified. As shown in Figure 1, NLRP3^−/−^ mice had a significantly higher larval burdens at 35 dpi compared with those of WT mice. Larval burden in Caspase 1/11^−/−^ and ASC^−/−^ mice did not significantly differ from those of WT mice. These results indicate that NLRP3 participates in the host-parasite interaction.

### NLRP3 played a role in *T. spiralis* -triggered immune response in vivo

**Figure 2:**
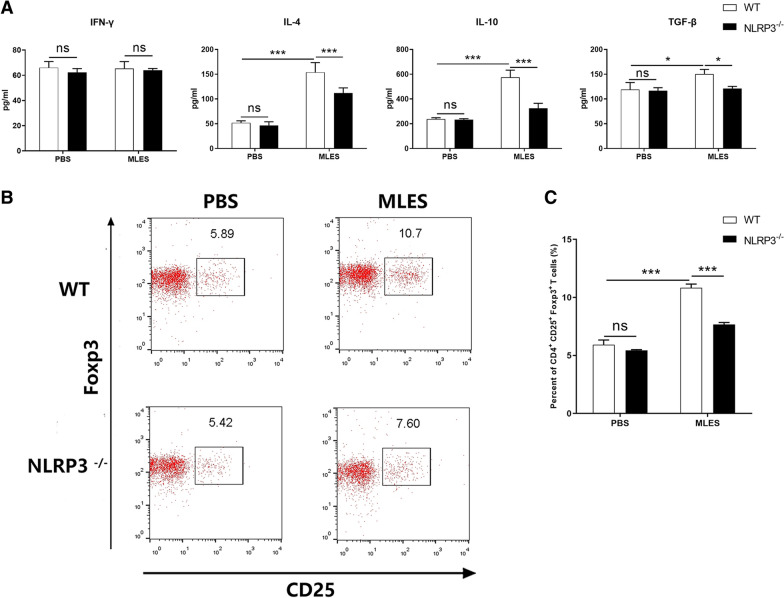
**NLRP3 played a role in** ***T. spiralis*** **-triggered immune response in vivo**. WT and NLRP3^−/−^ mice were injected intraperitoneally (i.p.) with phosphate-buffered saline (PBS) or MLES (100 μg) twice at 7 days intervals. Spleen cells were isolated 7 days after second injection. **A** IFN-γ, IL-4, IL-10 and TGF-β production was measured by ELISA. **B** Treg populations induced by MLES were measured. CD4^+^CD25^+^Foxp3^+^ T cells were determined by flow cytometry. The data represent the means ± standard deviations (SD) of each group (n = 6) of the results from three individual experiments *p < 0.05, **p < 0.01, and ***p < 0.001 as indicated by the line (one-way ANOVA with Tukey’s post-test). These figures are representative of three independent experiments.

To confirm the role of NLRP3 in the induction of immune response by *T. spiralis*, we measured cytokine production and Tregs population in WT and NLRP3^−/−^ mice injected with MLES. Our results demonstrated that MLES triggered the higher production of IL-4, IL-10 and TGF-β in WT mice than in NLRP3^−/−^ mice (Figure 2A). The level of IFN-γ was not significantly different among all groups. And MLES increased the percentage of CD4^+^ CD25^+^ Foxp3^+^ T cells compared to PBS in WT mice, whereas NLRP3^−/−^ mice treated with MLES exhibited lower Tregs populations compared to WT mice (Figure 2B, C).

### NLRP3 played a role in modulating the phenotype of MLES-treated DCs in vitro

**Figure 3:**
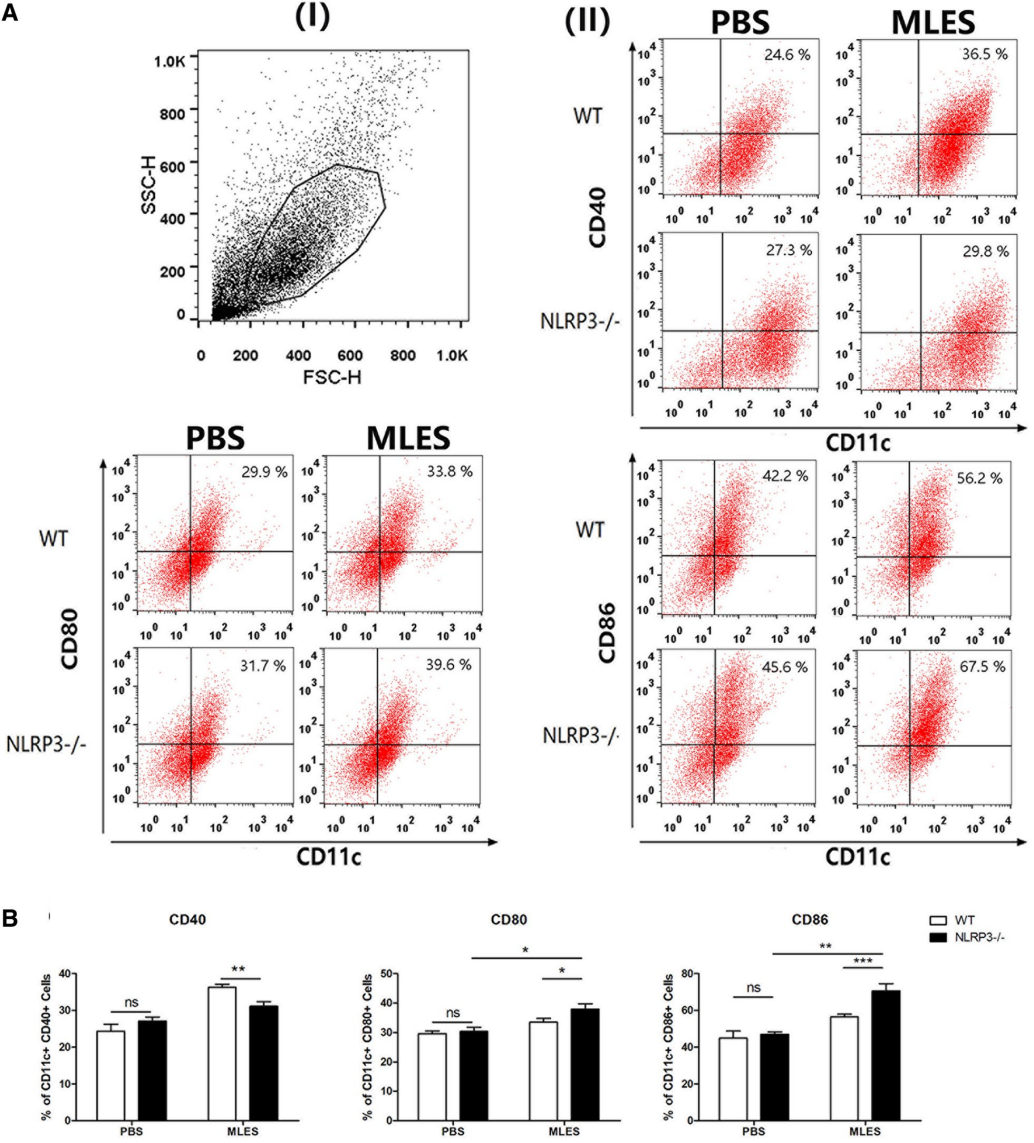
**The expression of CD40, CD80 and CD86 on DCs**. Wild type (WT) and NLRP3^−/−^ DCs were treated with MLES (50 μg/mL in vitro for 24 h. Immature DCs were stimulated with sterile phosphate-buffered saline (PBS) as a control. **A** (I) Representative dot plots for gating on viable cells. (II) The percentages of CD11c^+^ CD40^+^, CD80^+^ and CD86^+^ cells were measured by FACS. **B** The data represent the means ± standard deviations (SD) of each group (n = 3) of the results from three individual experiments *p < 0.05, **p < 0.01, and ***p < 0.001 as indicated by the line (one-way ANOVA with Tukey’s post-test). These figures are representative of three independent experiments.

**Figure 4:**
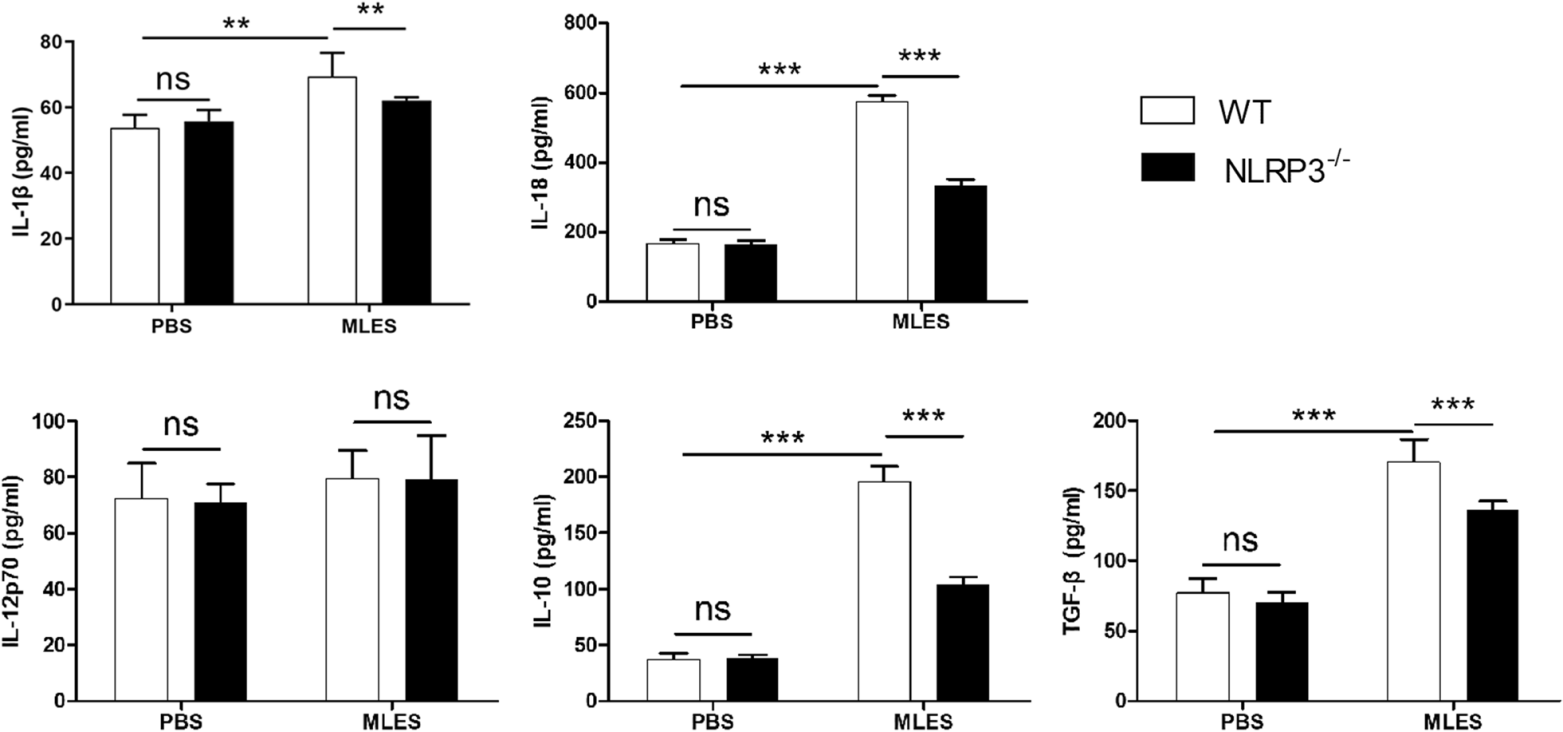
**Cytokine production by DCs.** Wild type (WT) and NLRP3^−/−^ DCs were treated with PBS or MLES (50 μg/mL) in vitro for 24 h. Cell culture supernatants were collected. IL-1β, IL-18, IL-12p70, IL-10 and TGF-β production was measured by ELISA. The data are the mean ± SD of each group (n = 3 from three independent experiments. *p < 0.05 and **p < 0.01 as indicated by the lines.

We showed that costimulatory molecules on the surface of DCs were upregulated by MLESs compared to those in the PBS group. NLRP3^−/−^ PBS-treated DCs displayed increased levels of CD40, CD80 and CD86 compared to those of WT DCs, whereas there is no significant difference. Notably, the percentage of CD11c^+^ CD40^+^ DCs from NLRP3^−/−^ mice treated with MLES was significantly inhibited compared to those of WT DCs treated with MLES (Figure 3). We demonstrated that MLESs promoted IL-1β, IL-18, IL-10 and TGF-β production compared to PBS group. These increases in production were diminished in DCs isolated from mice lacking NLRP3. However, no significant difference was observed in the level of IL-12p70 among the groups (Figure 4).

### NLRP3 in DCs played a role in the differentiation of Th2 cells and Treg responses in vitro

**Figure 5:**
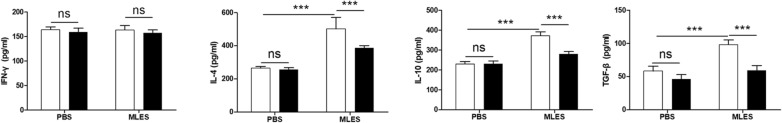
**Cytokine production in coculture of DCs and T cells in vitro**. DCs treated with PBS or MLESs and CD4^+^ T cells (1 × 10^6^/well) were cocultured with OVA (1 mg/mL) for 72 h. During the final 18 h, 2.5 μg/mL concanavalin-A (Con-A) was added. The supernatants of DC and CD4^+^ T cell cocultures were harvested for cytokine (IFN-γ, IL-4, IL-10 and TGF-β) analysis by ELISA. The results are shown as the mean ± SD (n = 3) of three different experiments. *p < 0.05, **p < 0.01, and ***p < 0.001 as indicated by the line (one-way ANOVA with Tukey’s post-test).

**Figure 6:**
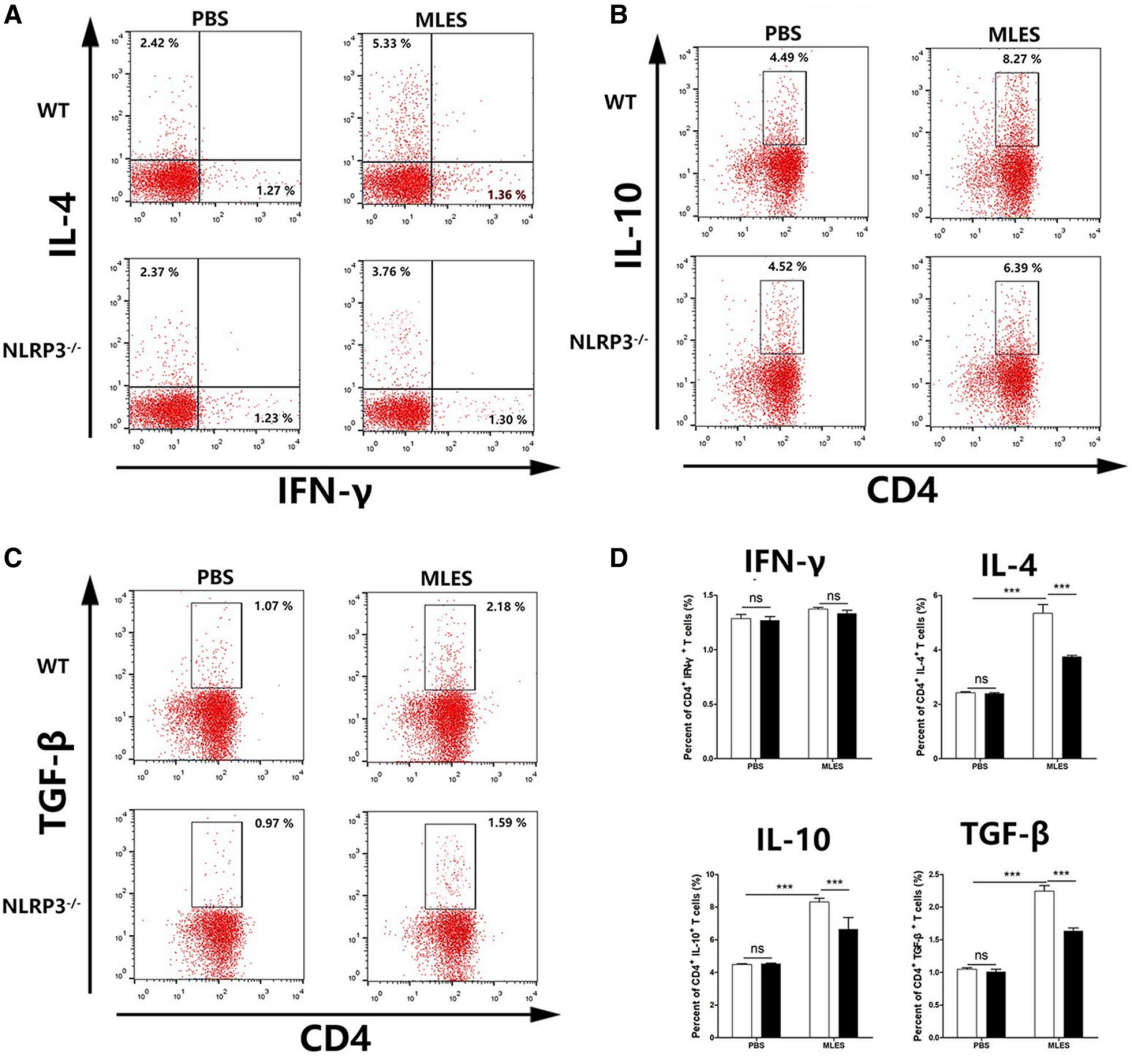
**Cytokine levels of CD4**^**+**^ **T cells by MLES –treated DCs in vitro.** For T helper-related cytokines expression, after coculture of DCs (1 × 10^5^/well) and CD4^+^ T cells (1 × 10^6^/well) for 72 h with OVA (1 mg/mL), then cells were incubated with 10 mg/mL Brefeldin A, 50 ng/mL phorbol 12-myristate 13-acetate (PMA) and 750 ng/mL Ionomycin for 6 h at 37 °C. **A** Percent of CD4^+^ IFN-γ^+^ T cells and CD4^+^ IL-4^+^ T cells were shown. **B** Percent of CD4^+^ IL-4^+^ T cells was shown. (B) Percent of CD4^+^ IL-10^+^ T cells was shown. **C** Percent of CD4^+^ TGF-β^+^ T cells was shown. **D** Data are shown as the mean ± SD (three independent experiments) of each group (n = 3). *p < 0.05, **p < 0.01, ***p < 0.001 as indicated by line (one-way ANOVA with Tukey’s posttest). These figures are representative of three independent experiments.

**Figure 7:**
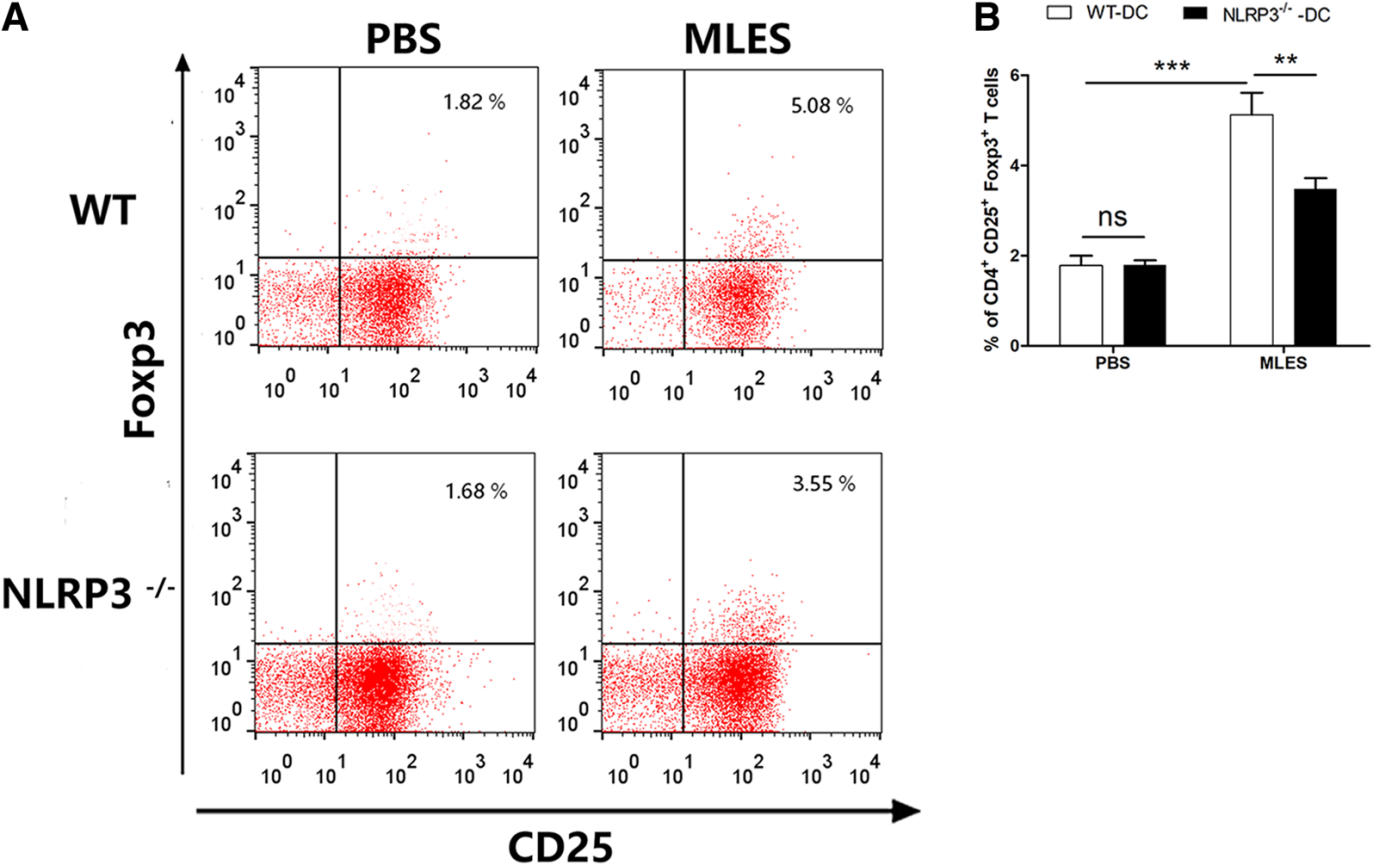
**Population of Treg cells by MLES-treated DCs in vitro. A** Treg populations induced by MLES –treated DCs were measured. CD4^+^CD25^+^Foxp3^+^ T cells were determined by flow cytometry, and **B** the summarized results are presented as the mean ± SD (n = 6) of three different experiments. *p < 0.05, **p < 0.01, and ***p < 0.001 as indicated by the line (one-way ANOVA with Tukey’s post-test). The figures are representative of three independent experiments.

MLESs lead to the production of Th2 cells and regulatory cytokines under the influence of DCs [19]. To investigate the role of NLRP3 in T cell differentiation by MLES-treated DCs, naïve CD4^+^ T cells were cocultured with WT or NLRP3 DCs. ELISA results showed that IFN-γ production induced by MLES-treated DCs from two mice failed to show a significant difference. WT MLES-treated DCs significantly increased IL-4, IL-10 and TGF-β secretion, which was significantly diminished when CD4^+^ T cells were cocultured with NLRP3^−/−^ DCs (Figure 5). Then the increased capacity of MLES-treated DCs for the induction of regulatory cytokines in T cells was measured by flow cytometry. MLES-DCs displayed an impaired potential to induce CD4^+^ IL-4^+^ T cells, CD4^+^ IL-10^+^ T cells and CD4^+^ TGF-β^+^ T cells in the absence of NLRP3 (Figure 6). Furthermore, FACS results showed that the population of CD4^+^ CD25^+^ Foxp3^+^ T cells was significantly increased by MLES-treated WT DCs compared to that of the PBS group, whereas NLRP3^−/−^ DCs induced a lower percentage of Tregs (Figure 7).

## Discussion

NLRs (nucleotide-binding domain leucine-rich-repeat-containing proteins) are known to be involved in pathogen-host interactions. The NLR family, pyrin domain containing 3 (NLRP3) is the most well-characterized NLRs and is important for immunity to diverse pathogens [20]. The role of NLRP3 in the host response to parasitic infection is just beginning to be understood but is comparatively less studied than that of bacterial or viral pathogens [21]. The NLRP3 have been shown to contribute to immune responses to infection with *Trypanosoma cruzi* [22], *Toxoplasma gondii* [23], *Paracoccidioides brasiliensis* [24], and *Leishmania* species [25], whereas the role of NLRP3 in the immune response to *Trichinella spiralis* infection is not yet clear. *T. spiralis* is a natural pathogen of rodents that establishes a chronic infection and results in impaired Th1 development and an immune bias towards Th2 cells or Tregs [6, 26]. Th2 and Treg responses are ultimately of critical importance for the survival of both the parasite and the host [27]. Our results demonstrated that *T. spiralis*-triggered Th2 and Treg response was decreased in NLRP3^/−^ mice treated with MLES, indicating that NLRP3 may participate in the development of Th2 and Treg response during *T. spiralis* infection.

Dendritic cells, as the orchestrators of immune responses, are well equipped to efficiently sense helminth infections in a variety of ways and trigger T cell responses by modulating DC phenotype and function [28]. In our study, we measured the phenotype of DCs treated with *T. spiralis* MLES, which can interact with host immune cells and induce an immune response that enables the survival of both the parasite and the host [6]. It has been reported that *T. spiralis* molecules modulate the phenotype of DCs via activation of TLR2 and TLR4 [7, 28], which are needed for NLRP3 activation [21]. There are other signals, such as K + efflux, ROS production, and lysosomal damage [21], and additional studies are needed to define the ways that *T. spiralis* MLESs activate NLRP3. Moreover, the NLRP3 inflammasome is a multiprotein assembly of the NLR-family protein NLRP3, pro-caspase-1, and an apoptosis-associated speck-like protein containing a CARD (ASC) adaptor, which is formed upon activation [25]. We demonstrated that larval burden increased in NLRP3^−/−^ mice but not in Caspase 1/11^−/−^ and ASC^−/−^ mice, suggesting that NLRP3 plays a role in the interaction between *T. spiralis* and the host.

We investigated the role of NLRP3 in modulating the DC phenotype. Interestingly, we observed that NLRP3 defects resulted in a reduction in the MLES-induced increased CD40 expression on the surface of DCs. The development of the Th2 response is impaired in the absence of CD40 on DCs [28].However, CD80 and CD86 expression on MLES-treated NLRP3^−/−^ DCs was enhanced compared to that of WT DCs. In contrast, activation of NLRP3 suppressed the expression of CD80 and CD86 [29]. Parasitic helminths are able to establish a state of immune hypo-responsiveness or tolerance by modulating the phenotype of DCs [30], and deletion of NLRP3 may influence this state. Furthermore, MLESs promoted IL-1β, IL-18, IL-10 and TGF-β production by WT DCs, while these elevated production levels were attenuated in MLES-treated NLRP3^−/−^ DCs. NLRP3 is critical for innate immunity and inducing the release of IL-1β and IL-18 [31]. NLRP3 inflammasome activation leading to IL-1β production is critical for the induction of a Th2 response [32]. IL-1β-deficient mice are susceptible to chronic *T. muris* infection and that the inability to expel the worms is associated with a defect in the development of a Th2 response, suggesting that the critical role of IL-1 in regulating Th2 response during gastrointestinal nematode infection [33]. IL-18 increases the production of IL-4 and IL-13 by T cells and enhances the Th2 response [34]. IL-4 and IL-13 levels decrease in NLRP3^−/−^ mice during intestinal stage of *T. spiralis* infection [35]. Additionally, NLRP3 inflammasome activation induces secretion of IL-18, which promotes Treg differentiation [36]. And DC-derived IL-18 acts directly on T cells to trigger their conversion to Tregs [37]. It has been reported that activation of NLRP3 leads to increased IL-10 production by DCs [38], and IL-10 has been linked to the induction of Th2 polarization [39]. Lack of NLRP3 impairs the immunomodulatory effect of TGF-β1 signalling [40], which is required for Treg differentiation [41]. Taken together, these results indicate that NLRP3 in MLES-treated DCs participates in the Th2 or Treg response. A balanced Th2 and Treg response is ultimately of critical importance for the survival of both the parasite and the host. DC, as the orchestrators of immune responses, are efficiently sense helminth infections in a variety of ways [27].

Coculture of DCs and naïve CD4^+^ T cells demonstrated that the levels of IL-4, IL-10 and TGF-β were significantly elevated by MLES-treated WT DCs, suggesting that CD4^+^ T cells may be polarized to Th2 cells and Tregs. The Th2 response with upregulation of IL-4 induced by *T. spiralis* promotes the development of eosinophils, mastocytosis, mast cell degranulation, and an immunoglobulin E (IgE) response that is associated with protection against *T. spiralis* infection [3].Treg response was involved in the anti-inflammatory effects of helminth infection and exist in the ML phase [42]. However, NLRP3^−/−^ DCs treated with MLESs induced reduced IL-4, IL-10 and TGF-β secretion in vitro. Consistently, FACS results showed that enhanced Treg populations were also inhibited in MLES-treated DCs lacking NLRP3. The level of IFN-γ was not altered, which may be due to the lack of significant changes in the levels of IL-12p70-producing DCs, which results in IFN-γ-secreting Th1 cell differentiation [43]. Another possible explanation is that Tregs prevent development of the Th1 response [38]. NLRP3 in DCs treated with MLESs strongly contributes to the induction of Th2 and Treg responses.

Taken together, these findings demonstrate that NLRP3^−/−^ mice are more susceptible to *T. spiralis* infection and present enhanced Th2 and Treg responses by modulating the DC phenotype via NLRP3. We identified for the first time the involvement of NLRP3 in host defences against *T. spiralis*. NLRP3 therefore represents an important target for the control of *T. spiralis* infection.

## Data Availability

The datasets supporting the conclusions of this article are included within the article.

## Abbreviations

ANOVA: one-way analysis of variance
ASC: apoptosis-associated speck-like protein containing a CARD
CD: cluster of differentiation
DC: dendritic cell
dpi: days post infection
IL: interleukin
IFN: interferon
IgE: immunoglobulin E
i.p.: intraperitoneally
MLES: muscle larvae excretory-secretory products
NLR: nod-like receptor
NLRP3: NLR family, pyrin domain containing 3
OVA: ovalbumin
PBS: phosphate-buffered saline
PPRs: pattern recognition receptors
TCR: T cell receptor
TGF: transforming growth factor
Th2: T helper 2
TLR: toll-like receptor
Treg: regulatory T cells
